# Comparison of Topical Hemostatic Agents in a Swine Model of Extremity Arterial Hemorrhage: BloodSTOP iX Battle Matrix *vs.* QuikClot Combat Gauze

**DOI:** 10.3390/ijms17040545

**Published:** 2016-04-12

**Authors:** Huixi Li, Lin Wang, Amjad Alwaal, Yung-Chin Lee, Amanda Reed-Maldonado, Taylor A. Spangler, Lia Banie, Reginald B. O’Hara, Guiting Lin

**Affiliations:** 1 Knuppe Molecular Urology Laboratory, Department of Urology, School of Medicine, University of California, San Francisco, CA 94143, USA; huixi.li@ucsf.edu (H.L.); lin.wang@ucsf.edu (L.W.); amjadwal@yahoo.com (A.A.); leeyc12345@yahoo.com.tw (Y.-C.L.); amanda.reed-maldonado@ucsf.edu (A.R.-M.); lia.banie@ucsf.edu (L.B.); 2 VDx Veterinary Diagnostics, Davis, CA 95616, USA; tspangler@vdxpathology.com; 3 Department of Aeromedical Research, U.S. Air Force School of Aerospace Medicine, Wright-Patterson Air Force Base, Dayton, OH 45433, USA; reginald.ohara@yahoo.com

**Keywords:** hemostatic agents, BloodSTOP Battle Matrix, QuikClot Combat Gauze, hemorrhage control, swine

## Abstract

BloodSTOP iX Battle Matrix (BM) and QuikClot Combat Gauze (CG) have both been used to treat traumatic bleeding. The purpose of this study was to examine the efficacy and initial safety of both products in a swine extremity arterial hemorrhage model, which mimics combat injury. Swine (37.13 ± 0.56 kg, N_BM_ = 11, N_CG_ = 9) were anesthetized and splenectomized. We then isolated the femoral arteries and performed a 6 mm arteriotomy. After 45 s of free bleeding, either BM or CG was applied. Fluid resuscitation was provided to maintain a mean arterial pressure of 65 mmHg. Animals were observed for three hours or until death. Fluoroscopic angiography and wound stability challenge tests were performed on survivors. Tissue samples were collected for histologic examination. Stable hemostasis was achieved in 11/11 BM and 5/9 CG subjects, with recovery of mean arterial pressure and animal survival for three hours (*p* < 0.05, Odds Ratio (OR) = 18.82 (0.85–415.3)). Time to stable hemostasis was shorter for the BM-treated group (4.8 ± 2.5 min *vs.* 58 ± 20.1 min; Median = 2, Interquartile Range (IQR) = 0 min *vs.* Median = 60, IQR = 120 min; *p* < 0.05) and experienced longer total stable hemostasis (175.2 ± 2.5 min *vs.* 92.4 ± 29.9 min; Median = 178, IQR = 0 min *vs.* Median = 120, IQR = 178 min; *p* < 0.05). Post-treatment blood loss was lower with BM (9.5 ± 2.4 mL/kg, Median = 10.52, IQR = 13.63 mL/kg) compared to CG (29.9 ± 9.9 mL/kg, Median = 29.38, IQR = 62.44 mL/kg) (*p* = 0.2875). Standard BM products weighed less compared to CG (6.9 ± 0.03 g *vs.* 20.2 ± 0.4 g) (*p* < 0.05) and absorbed less blood (3.4 ± 0.8 g *vs.* 41.9 ± 12.3 g) (*p* < 0.05). Fluoroscopic angiography showed recanalization in 5/11 (BM) and 0/5 (CG) surviving animals (*p* = 0.07, OR = 9.3 (0.41–208.8)). The wound stability challenge test resulted in wound re-bleeding in 1/11 (BM) and 5/5 (CG) surviving animals (*p* < 0.05, OR = 0.013 (0.00045–0.375)). Histologic evidence indicated no wound site, distal limb or major organ damage in either group. BM is more effective and portable in treating arterial hemorrhage compared to CG. There was no histologic evidence of further damage in either group.

## 1. Introduction

Military trauma, which is largely caused by high-energy projectile and improvised explosive devices, contrasts significantly from civilian trauma with respect to mechanism and injury pattern. Despite substantial advances in hemostatic technologies over the past two decades, catastrophic hemorrhage from direct large-vessel damage is the leading cause of potentially preventable death of combat casualties [1]. The natural rapid clotting ability of blood is insufficient to control bleeding during severe large vessel injury; clotting may also be diminished due to massive tissue trauma and acute coagulopathy [2]. Bleeding significantly impacts morbidity and mortality due to prolonged hypotension, lack of coagulation factors, and multiple organ failure [1,2,3].

Currently, there is no licensed hemostatic modality to treat non-compressible (internal) hemorrhage. Rapid control of bleeding from compressible (external) wounds is vital [4,5]. Hemostatic dressings are generally used for compressible vascular injuries for wounds inappropriate for tourniquets, such as those in the groin, neck, or axilla [6]. Many dressings have been tested for this purpose, including chitosan-based dressings [4,7], porous polyethylene fiber-based dressings, hydrogels [8], fibrinogen/thrombin-impregnated matrices [9,10] and kaolin-impregnated gauze [1]. Kaolin-impregnated gauze is the only agent associated with 0% mortality in a swine model of lethal groin injury, but the gauze produced significant side effects, including rapid fluid absorption, exothermic reaction, and leaching into tissue [11,12,13]. A literature review shows that an ideal hemostatic dressing has yet to be developed.

**Figure 1:**
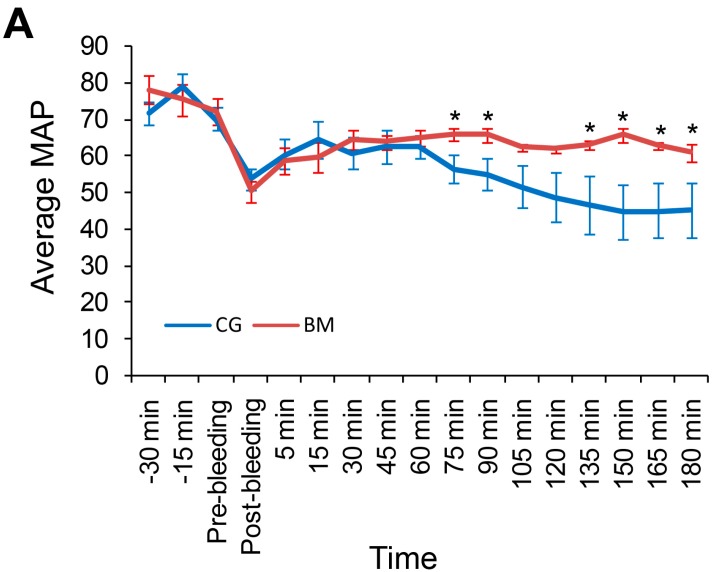

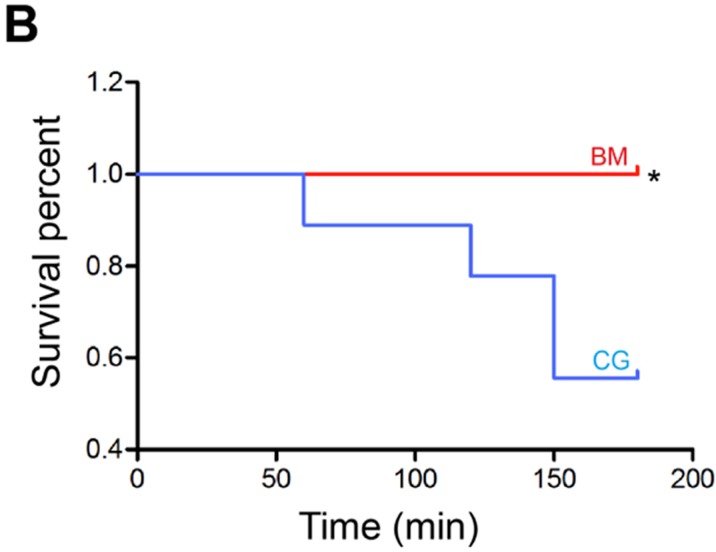
Mean arterial pressure (MAP) and survival rate. (**A**) The mean MAP for each swine group (surviving and non-surviving). The MAP of BloodSTOP iX Battle Matrix (BM)-treated animals significantly increased compared to that of QuikClot Combat Gauze (CG)-treated animals in response to resuscitation at 75, 90, and 135–180 min (* *p* < 0.05) post-treatment; (**B**) Log-rank (Mantel–Cox) analysis of survival time of swine treated with each dressing. The BM-treated swine lived significantly longer compared to CG-treated swine (* *p* < 0.05).

BloodSTOP iX Battle Matrix (BM, LifeScience PLUS, Mountain View, CA, USA, see Figure 1A) is a biocompatible, woven fiber matrix made from regenerated cotton cellulose. When it contacts liquid, BM adheres to the wound, initiates blood coagulation, stops hemorrhage, and creates a protective coating, thereby creating an optimal wound healing milieu. Its water solubility allows for its easy removal by rinsing without disruption of the clotted surface. Its composition actually provides advantages for hemostasis and improved wound management and patient comfort compared to traditional chemical powders, sprays, adhesive bandages, or other animal-based products [14]. In our previous study, BM was more effective in reducing bleeding time compared to Surgicel in a rat model of partial nephrectomy. In an aortic needle injury model, BM achieved hemostasis faster than Gelfoam or Surgicel [15].

In the current study, the efficacy of BloodSTOP iX Battle Matrix was compared to standard kaolin-impregnated gauze (QuikClot Combat Gauze, CG), which has been recommended as the first-line treatment for those life-threatening hemorrhages [16].

## 2. Results

### 2.1. Biological Characteristics of Animals for in Vivo Experiment

Swine in both groups had similar body weight (36.9 ± 0.5 kg *vs.* 37.4 ± 1.1 kg, *p* > 0.05). No significant differences were noted in baseline physiologic or hematological measurements (Table 1). Final values for hematological measurements at the conclusion of the experiments are shown in Table 2. The HGB (hemoglobin), HCT (hematocrit), PLT (platelets), PT (prothrombin time) and aPTT (activated partial prothromboplastin time) corresponded to the degree of blood loss and fluid replacement in each group. Animals in the BM group had significantly higher HGB and HCT values compared to CG-treated animals (*p* < 0.05). Animals in the BM group also had shorter PT and aPTT times (*p* < 0.05).

### 2.2. Hemostasis Achievements and Blood Loss

The incidence of initial hemostasis achievement (secure hemostasis for at least three minutes immediately after the first product application) was similar between groups (36.4% (10.93%–69.21%), 4/11 for BM and 44.44% (13.7%–78.9%), 4/9 for CG). The mean number of applications was two for both groups. The baseline weight of the BM product was significantly less compared to the CG product (6.9 ± 0.03 g *vs.* 20.2 ± 0.4 g, *p* < 0.05), and BM also absorbed significantly less blood compared to CG (3.4 ± 0.8 g *vs.* 41.9 ± 12.3 g, *p* < 0.05).

BM-treated animals showed significantly shorter times to hemostasis (4.8 ± 2.5 min *vs.* 58 ± 20.1 min, *p* < 0.05) (Median = 2, Interquartile Range (IQR) = 0 min *vs.* Median = 60, IQR = 120 min, *p* < 0.05) and longer total stable hemostasis time (175.2 ± 2.5 min *vs.* 92.4 ± 29.9 min, *p* < 0.05) (Median = 178, IQR = 0 min *vs.* Median = 120, IQR = 178 min, *p* < 0.05). The mean time to bleeding control by those dressings (stable hemostasis time) and other hemostatic outcomes are shown in Table 2.

The mean pre-treatment blood loss was 6.8 ± 0.6 mL/kg in the BM group and 8.3 ± 0.9 mL/kg in the CG group (Table 2, *p* > 0.05). The post-treatment blood loss in BM-treated animals (9.5 ± 2.4 mL/kg, Median = 10.52, IQR = 13.63 mL/kg) was less compared to that of CG-treated animals (29.9 ± 9.9 mL/kg, Median = 29.38, IQR = 62.44 mL/kg, Table 2, *p* = 0.2875).

### 2.3. Mean Arterial Pressure and Survival

The hemostatic condition of the swine within the experiment is shown as mean MAP in Figure 1A. Baseline pressures (Table 1) decreased sharply post injury, and hemorrhage did not differ significantly between groups. The MAP significantly differed at 75, 90, and 135–180 min after injury for animals treated with BM compared to those treated with CG (*p* < 0.05), with the animals in the BM group showing MAP improvement after injury.

Animals in the BM group survived longer compared to those in the CG group (180 ± 0 min *vs.* 150.4 ± 14.0 min, Table 2). All 11 (100%, 71.51%–100%) BM-treated and 5/9 (55.6%, 21.3%–86.4%) CG-treated animals lived for the entire experiment (Figure 1B, *p* < 0.05) with final MAPs of 60.7 ± 2.3 mmHg and 45.1 ± 7.5 mmHg, respectively (Table 1, *p* < 0.05).

### 2.4. Fluoroscopic Angiography

**Figure 2:**
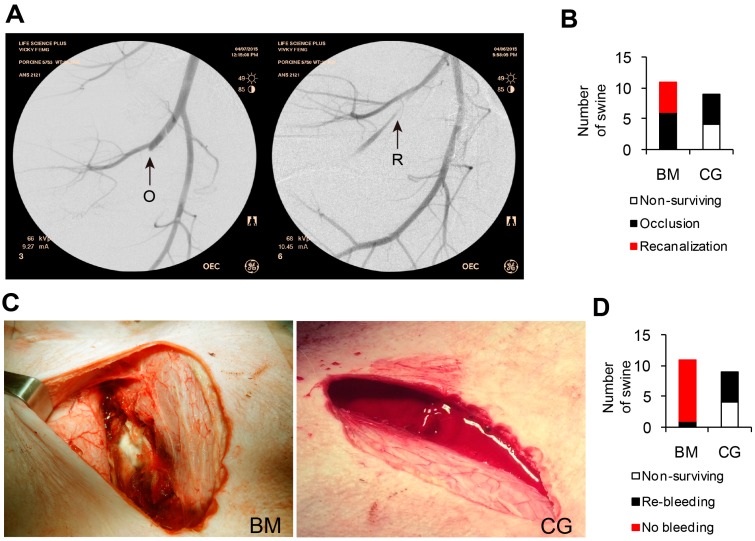
Angiogram of surviving animals and Morphological assessment after removal of tested materials. The femoral artery was occluded at the injury site in 100% of animals treated with CG (5/5) and in 54.5% of animals treated with BM (6/11). In 45.5% (5/11) of animals treated with BM, the artery was narrowed at the injury site but blood was present in the distal femoral artery. Video angiogram analysis demonstrated anterograde flow (not retrograde from collateral circulation). After CG removal, 100% of hemostatic clots ruptured and re-bleeding occurred. In contrast, 90.9% of injuries treated with BM demonstrated stable hemostatic clots with sticky gels over the arterial injury site. (**A**) Representative images from fluoroscopic angiography. The arrow indicates the artery injury site. O: occlusion; R: recanalization; (**B**) Number of animals with injured artery recanalization or occlusion; (**C**) Representative images of injury site after product removal; (**D**) Number of animals with or without re-bleeding after product removal.

Fluoroscopic angiography was performed through the cannulated right carotid artery to check arterial blood flow and vascular structures of the injured leg in both the BM and CG groups. The results demonstrated successful blockage of blood flow at the treated site of femoral arteries in 100% (47.8%–100%, 5/5) of surviving animals in the CG group (Figure 2A,B). Recanalization occurred in 45.5% (16.8%–76.6%, 5/11) of surviving animals in the BM group. Partial blood flow blockage in femoral arteries at the BM-treated sites allowed for distal blood flow through the injury sites.

### 2.5. Morphological Assessment

The simulated walking test caused no bleeding in either group. After removal of the laparotomy gauze and products, re-bleeding occurred in 9.1% (0.23%–41.3%, 1/11) of BM animals and 100% (47.8%–100%, 5/5) of CG animals (Figure 2C,D). CG dressings were easily removed from wounds but resulted in rupture of the hemostatic clot and re-bleeding at the injury site. After removal of the top BM layers, an adherent gel remained over the site of arterial injury and surrounding tissue, resulting in a very strong, solid, and stable hemostatic clot. This adherent material served to create a safe and effective “seal membrane” (Figure 2C).

Fluoroscopic angiography and morphological assessment showed that BM performed better in achieving stable hemostasis and maintaining blood supply to the injured limb.

### 2.6. Histologic Assessment

BM is a natural, bio-absorbable, cellulose matrix that readily dissolves in water (as well as formalin fixative used for tissue fixation). As such, the test article was generally dissolved and not readily apparent in histological sections. In most cases, the test article left a “void” or “clear space” in the tissue section where it had been deposited. Occasionally, additional subtle, microscopic evidence of the test article was discernible around the treatment site. Histologically, the test article was acellular, mostly amorphous (or occasionally arranged into threads) with faintly basophilic or eosinophilic (amphophilic) staining properties. Frequently, there were clusters of coagulated erythrocytes and platelets enmeshed in the voided space left by the test article. Any of these features were interpreted as “evidence” that the test article was present in the histologic section. The control article consisted of a micro-particulate material (kaolin) impregnated in a polyester/rayon gauze. The impregnated gauze was removed at the end of the procedure. No evidence of micro-particulate material was found in histologic sections.

**Figure 3:**
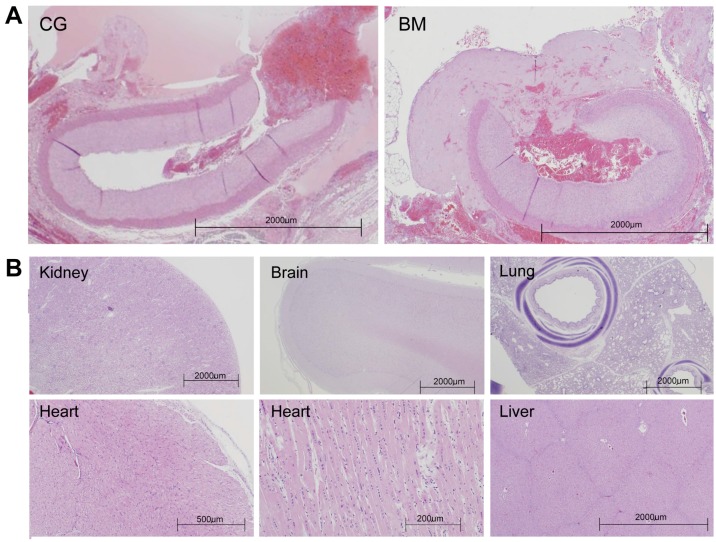
Histological assessment using hematoxylin and eosin (H&E) staining. (**A**) Low-power magnification of the arterial test segment demonstrating arteriotomy. The artery lumen contains clumping platelets bound by fibrin as well as red blood cells. A “seal membrane” was noted in the BM group; (**B**) Representative section of kidney, brain, lung, heart, and liver tissues.

Typically the arterial sections contained some degree of acute thrombus material that varied slightly in character and density. Presence of intraluminal thrombus was most often associated with some degree of endothelial denudation or reactivity, a feature that may be associated with vascular clamping, creation of the arteriotomy, or compression. Interestingly, BM formed a “sealed membrane” structure overlapping the injured arteries (Figure 3A).

The perivascular anatomy was evaluated histologically. Acute pathological changes associated with the vascular cut down, three-hour wait time, and other study procedures were anticipated and interpreted as “normal”. Most often the treated vascular area was suffused with hemorrhage and early neutrophilic inflammation. The femoral vein, femoral nerve, and surrounding musculature were not associated with any pathological changes attributable to the application of the test or control article. There was no indication of embolic test or control material in distal tissues. Furthermore, end-organ and distal-tissue analysis was mostly unremarkable with no evidence of thromboembolism of test or control material (Figure 3B).

## 3. Discussion

Battlefield injuries and some civilian trauma caused by industrial disasters or vehicular accidents present complex, irregular wounds with similar patterns that require quick hemorrhage control. Extremities are the most common injury sites among trauma patients and military casualties [2]. In this study, the animal model represented a severe groin wound with bleeding, for which application of regular gauze in inadequate and for which tourniquet application is impossible [1,17]. This is a challenging and difficult bleeding condition for which the ideal dressing has yet to be identified. In an attempt to accurately test the efficacy of the BM product, we have minimized all accessory factors that would aid spontaneous hemostasis in our experiment. Most uncontrolled bleeding-associated deaths occur within the first hour after injury [16]. Therefore, the post-application observation time in our experiment was set to three hours. Although the bleeding was not consistently arrested by dressings, it was ameliorated enough to allow longer survival, up to three hours, mimicking pre-hospital time for soldiers injured on the battlefield.

Currently, two hemostatic dressings that have gained market prominence are Celox, by Medtrade, and QuikClot Combat Gauze (CG), by Z-Medica, the comparator product in this study. Both of these dressings are made of common gauze with an impregnated component. Celox is gauze with chitosan granules, QuikClot CG is gauze with kaolin mineral powder. Chitosan is a weak hemostatic agent, a product of shellfish endoskeleton that has been known to cause an allergic response in some patients. Kaolin triggers the coagulation cascade, but is an inert, nonbiodegradable mineral powder that collects in the wound, requiring thorough and tedious cleaning of the wound after use. Neither is water soluble, and both can cause rebleeding upon removal. Celox, CG and BM are all comparably priced.

CG has demonstrated effectiveness in multiple studies and is currently the hemostatic dressing deployed with the United States military. To be an effective hemostatic agent, the hemostatic function of CG depends on blood clotting capacity, its efficacy may be affected by the coagulation status of the blood; therefore, it may be less effective in coagulopathic patients. In previous reports, CG-treated animal showed a survival rate ranging from 50% to 100% [18,19,20]. In our current experiment, the survival rate in CG treated animals was 55.6%. CG treatment produced hemostasis in 5 out of 9 swine. Hemostatic function in surviving animals was mediated through enhanced blood-clotting and the development of hemostatic clots in conjunction with the gauze. For animals failing treatment in the CG group, the dressing seemed capable of stopping low-pressure arterial bleeding. Upon fluid administration with return of baseline blood pressure, the dressing failed and re-bleeding resulted in exsanguination. Further fluid administration diluted the blood and formation of a second clot was difficult to achieve. The dilution of coagulation factors and platelets is an important cause of coagulopathy in massively resuscitated trauma patients [21]. In fact, resuscitation practices have been challenged for a long time [9,22]. Because massive hemorrhage can rapidly progress into a difficult-to-manage or life-threatening situation, interventions are usually applied to compensate for platelet and coagulation factor loss. In this experiment, a large amount of crystalloid (lactated Ringer’s solution) was used for resuscitation when hemostasis was unsuccessful to maintain the target blood pressure, thereby increasing the likelihood of clot failure and re-bleeding. Over-dose resuscitation resulted in considerable tissue edema and occasional death, likely related more to excessive hemodilution rather than to blood loss alone. Based on these findings, survival in CG-treated animals was likely diminished by the ongoing fluid administration.

We are encouraged by the performance of BM in this study. BM is lighter and more portable, and its application was associated with significantly less blood loss, improved physiological/hematological indices, shorter time to hemostasis, longer and more stable bleeding control, and higher recanalization rate. These features allowed for better MAP maintenance and an improved survival rate compared to CG-treated animals.

The gel seal and the tenacity of the clot formed after application of BM might both be advantages over CG. Those properties show promise in allowing evacuation of the injured warrior without disruption of the clot. The robustness of the clot will also allow for more measured surgical treatment at higher echelons of care. The ability of BM gel to absorb and hold water in its network structure also acts as a moist dressing and could absorb and retain the wound exudates along with bacteria and other foreign bodies. All of these features help to maintain a micro-climate for biosynthetic reactions on the wound surface necessary for cellular activities, including fibroblast proliferation and keratinocyte migration [8]. More importantly, BM restored distal blood flow in the injured limbs through its sealant properties that are independent of continuous external compression. This property could potentially increase the limb salvage rate in patients with severe non-compressible, vascular injuries.

The physical characteristics and gauze type may also affect results. The larger CG size is an advantage that allows packing large complex wound with complete coverage of the injured tissue or multiple bleeding sites. In our experiment, physical characteristics also affected packing time. Packing time is an important parameter for the application of gauze in the battlefield [5]. We did not record the packing time in this project. However, according to the procedure, more time may be required to pack BM compared to CG, and this should be studied further.

According to the pathological assessment, there were no significant differences between the products. No vital abnormal changes were found in either BM- or CG-treated animals. It remains to be demonstrated whether CG is associated with long-term side effects that have been found with other granular hemostatic products [23,24]. In addition, BM dressing use also requires long-term survival studies. Battlefield hemorrhage is usually complicated by other conditions, including hypothermia and acidosis, which were not addressed by this experiment [9]. CG has been tested thoroughly under some extreme physiologic conditions, including acidotic and coagulopathic conditions [25]. Future directions should include conducting BM testing in more challenging models and modifying products to facilitate handling and application, even by inexperienced persons.

## 4. Materials and Methods

### 4.1. Test Materials

This study was approved by the Institutional Animal Care and Use Committee (IACUC) of PMI Preclinical LLC (San Carlos, CA, USA; permission code: ANS-2121, IAC#1779; 4 March 2015). The animal model utilized in the study has been widely used to demonstrate the efficacy of hemostatic dressings and is recognized by the US Army Institute of Surgical Research and the US Food and Drug Administration (FDA) as the benchmark model for assessing hemostatic efficacy for severe bleeding.

All test materials have received FDA clearance as safe devices for temporary external use to control moderate to severe bleeding. CG is a product of the Z-Medica Corporation (Z-MEDICA, LLC, Wallingford, CT, USA). It is a vacuum-packed and Z-folded dressing made of a 3” × 144” Z-folded, 48 layer medical gauze impregnated with a contact (intrinsic) pathway-activating clotting agent known as kaolin [26]. BM is a product of LifeScience PLUS (LifeScience PLUS, Inc., Mountain View, CA, USA). It is a Z-folded 3” × 24”, 8 layer dressing made from etherified, oxidized, regenerated cotton cellulose. The material is water-soluble and forms a sticky gel when it contacts blood or other fluids.

### 4.2. Animal Preparation

Yorkshire cross-bred swine (*Sus scrofa*, female, 37.13 ± 0.56 kg, *n* = 20) were purchased from Pork Power Farms (Turlock, CA, USA) for this study. Swine were fasted for 12 to 18 h with water provided *ad libitum* before surgery. Animals were pre-medicated with buprenorphine (0.025 mg/kg intramuscularly (IM)) for analgesia and glycopyrrolate (0.01 mg/kg, IM) to reduce secretion of saliva and block bradycardia mediated by the vagus nerve. Then, injection of tiletaminezolazepam (Telazol, 4 mg/kg, IM) was used for induction and 5% isoflurane in oxygen via facemask used for initial anesthesia. The swine were intubated and ventilated with 100% oxygen. The tidal volume and ventilation rate were adjusted to maintain an end tidal *P*_CO2_ of 40 ± 2 mmHg. Anesthesia was maintained with 2% isoflurane added to 100% oxygen gas via respirator. Lactated Ringer’s (LR) was administered at 5 mL/kg/h through an ear vein. Blood samples, blood pressure (systolic and diastolic), mean arterial pressure (MAP), and heart rate were collected or recorded during the experiment through cannulation of the right carotid artery. The right jugular vein was catheterized for administration of resuscitation fluid. No heparin was administered during the procedure. The blood collection bottle was placed on a scale to measure blood loss in grams.

### 4.3. Surgical Procedures

The surgical procedures and treatments were developed to follow the recognized animal model, as previously reported [1]. A midline laparotomy was performed to expose the spleen and bladder. Splenectomy was then performed to reduce variation resulting from splenic auto-transfusion. The blood loss from splenectomy was substituted with a volume of LR equivalent to three times the spleen’s weight. A Foley catheter was placed through a cystotomy to drain urine during surgery. The abdomen was closed with sutures. Preinjury (baseline) blood samples were collected to measure hemoglobin (HGB), hematocrit (HCT), prothrombin time (PT), activated partial prothromboplastin time (aPTT), platelet (PLT), fibrinogen, pH, lactate, base excess, complete blood counting (CBC), and blood chemistry.

An incision of approximately 10 cm was made in the inguinal region. After removing the abductor muscle to expose the femoral artery, hemorrhage was induced by dissecting 5 cm of artery free from the adjacent tissue. Injury to the adjacent femoral vein and nerve was avoided. To prevent vasospasm and to dilate the artery to its normal caliber, the artery was bathed with gauze soaked with 3 milliliters of 2% lidocaine for 10 min. A stable MAP ≥ 60 mmHg was ensured prior to arteriotomy. Next, maintenance fluids were withdrawn and the artery was clamped proximally and distally with bulldogs. A 6 mm diameter arteriotomy was made on the anterior vessel surface using a vascular punch (International Biophysics Corp., Austin, TX, USA). The bulldog clamps were then released, and free bleeding was allowed for 45 s. Pre-treatment blood loss was measured to be the summation of blood suctioned into pre-weighed canisters and blood absorbed by pre-weighed dressings.

The products were applied according to the manufacturers’ instructions, and surgeons were blinded to test materials until the time of application. After 45 s of free bleeding, BM or CG was packed into the wound. A laparotomy sponge was immediately placed over the product, pressing against the wound with sufficient and equal pressure to try to stop blood loss.

Resuscitation began after 30 s of compression; initially, 150 mL of colloid was administered. Pre-treatment blood loss was weighed and, if it exceeded 150 mL, an additional colloid was administered to match the additional blood volume lost. The colloid was administered at approximately 100 mL/min intravenously. At this point, maintenance crystalloid fluids were added. The target of resuscitation was to raise the MAP to 65 mmHg, the average baseline blood pressure of anesthetized pigs, in order to increase the performance demands of the dressings, which made it suitable for dressing selection in extreme conditions, such as in the battlefield. Compression was stopped after 2 min and hemostasis was observed for three minutes with the laparotomy gauze in place. If re-bleeding occurred during this period, the laparotomy gauze and the failed agent were removed, and they were replaced with fresh product and fresh laparotomy gauze. The two-minute compression was repeated and followed by another three-minute observation. At most, wounds were treated 3 times with each product, irrespective of final hemostatic outcome. For the next 3 h, with all materials in place, hemostasis was then observed at 30 min intervals. Any shed blood and blood absorbed by the dressing or laparotomy sponge during this period was collected and measured as post-treatment blood loss. A target MAP of 65 mmHg was achieved throughout the experiment by administering a maximum of 10 L of LR. The above resuscitation procedure is comparable to the model used to test the efficacy of CG identified by FDA.

### 4.4. Endpoints

Monitoring continued for up to three hours or until animal death, defined as MAP < 20 mmHg and end tidal *P*_CO2_ < 15 mmHg. Blood samples were collected for hematological measurements before euthanizing the animals. After 3 h, the surviving animals underwent fluoroscopic angiography. Arterial blood flow and vascular structures were assessed in both the injured and contralateral legs. Through the cannulated right carotid artery, a catheter was guided down the aorta to the bifurcation and then the angiogram was performed. Next, the treated legs of the surviving swine were flexed and stretched five times simulating a walking condition to test the stability of the hemostasis provided by the test products. At the conclusion of these tests, the laparotomy sponge and product were removed from the wound assessed for injury and vessel patency. Photographs were obtained.

### 4.5. Gross Pathology and Histopathology

Animals were then humanely euthanized and subjected to a thorough necropsy examination by a Board Certified Veterinary Pathologist. Representative tissue samples collected for histologic examination included the injured artery, adjacent femoral vein, muscle tissues, soft tissue of distal extremity supplied by the treated artery, femoral vein of the contralateral leg (as a control), and major organs (kidney, liver, heart, lung, brain).

Formalin-fixed tissue samples were hand-carried and shipped to VDx Company (Davis, CA, USA) for histology processing. All slides were reviewed by the pathologist, who was blinded to treatment group. Histopathological analysis of the treatment site included a comment on the presence or absence of the test article, as well as any vascular pathology (endothelial disruption, injury to the internal elastic lamina, tunica media, external elastic lamina or adventitia, and assessment of the surrounding musculature). End-organ analysis included an assessment for any pathology, particularly any evidence of micro-emboli from either product.

### 4.6. Data Analysis

Data are expressed as means ± standard of error of the mean (SEM) for parametric test and Median with IQR for nonparametric test. The categorical variables were calculated using Chi-square test. A student’s *t*-test (parametric) or Mann–Whitney test (nonparametric) was used to compare the means of continuous variables between two groups. Survival rates were compared using Log-rank (Mantel–Cox) test. *p* < 0.05 was considered statistically significant. Analysis was completed using Prism 4 (GraphPad Software, San Diego, CA, USA).

## 5. Conclusions

In this study, the BM hemostatic dressing demonstrated superiority in achieving hemostasis in this extremity arterial hemorrhage swine model compared to CG. The effect was durable, as BM was able to maintain hemostasis longer. The ability to effectively control and sustain stable hemostasis and MAP without the need for excessive resuscitation (and maintenance of distal blood flow in some cases) supports the use of this dressing as a novel hemorrhage control tool to enhance traumatic injury outcomes in both civilian and military settings. By all measures, BM better controlled hemorrhage and averted death in this model of extremity arterial hemorrhage compared to CG.

## Figures and Tables

**Table 1:** Physiological and hematological measurements pre-treatment and post-treatment.

Parameter	Pre-Treatment				Post-Treatment			
Measure	BM (*n* = 11)		CG (*n* = 9)		BM (*n* = 11)		CG (*n* = 9)	
Temperature (°C)	36.1	±0.3	36.3	±0.4	36.9	±0.5	36.4	±0.5
MAP (mmHg)	78.3	±3.7	70.4	±3.1	60.7	±2.3 *	45.1	±7.5
HGB (g/dL)	9.1	±0.3	8.8	±0.3	7.1	±0.4 *	4.7	±1.0
HCT (%)	27.9	±0.9	27.3	±0.8	21.6	±1.2 *	14.7	±3.1
PLT (1000/µL)	334.5	±21.2	340.3	±20.4	229.5	±9.9	182.2	±36.6
PT (s)	13.9	±0.2	14.2	±0.2	14.9	±0.2 *	22.0	±3.6
aPTT (s)	13.9	±0.4	14.8	±0.3	14.8	±0.4 *	18.9	±1.7
Fibrinogen (mg/dL)	153.9	±7.7	201.3	±58.3	111.7	±7.5	87.2	±12.2
Lactate (mM)	16.7	±1.7	18.9	±2.2	19.8	±2.8	54.3	±18.5

Data expressed as means ± SEM and analyzed by *t*-test. * *p* < 0.05 compared to CG. MAP, mean arterial pressure; HGB, hemoglobin; HCT, hematocrit; PLT, platelets; PT, prothrombin time; aPTT, activated partial prothromboplastin time; BM, BloodSTOP iX Battle Matrix; CG, QuikClot Combat Gauze.

**Table 2:** Treatment outcomes using BM or CG following groin arterial hemorrhage.

Outcome	BM (*n* = 11)		CG (*n* = 9)	
Pre-treatment blood loss (mL/kg)	6.8	±0.6	8.3	±0.9
Post-treatment blood loss (mL/kg)	9.5	±2.4 *	29.9	±9.9
Total resuscitation fluid (mL/kg)	93.4	±16.4	110.0	±22.8
Time to stable Hemostasis (min)	4.8	±2.5 *	58.0	±20.1
Total stable hemostasis time ^†^ (min)	175.2	±2.5 *	92.4	±29.9
Survival time (min)	180.0	±0.0	150.4	±14.0

Data expressed as means ± SEM and analyzed by *t*-test (except survival time). * *p* < 0.05 compared to CG. ^†^: Total stable hemostasis time was the time from hemostasis achievement to the end of the three-hour observation or until animal death.
